# *De novo* and rare inherited mutations implicate the transcriptional coregulator TCF20/SPBP in autism spectrum disorder

**DOI:** 10.1136/jmedgenet-2014-102582

**Published:** 2014-09-16

**Authors:** Christian Babbs, Deborah Lloyd, Alistair T Pagnamenta, Stephen R F Twigg, Joanne Green, Simon J McGowan, Ghazala Mirza, Rebecca Naples, Vikram P Sharma, Emanuela V Volpi, Veronica J Buckle, Steven A Wall, Samantha J L Knight, International Molecular Genetic Study of Autism Consortium (IMGSAC), Jeremy R Parr, Andrew O M Wilkie

**Affiliations:** 1 Weatherall Institute of Molecular Medicine, John Radcliffe Hospital, Oxford, UK; 2 NIHR Biomedical Research Centre, Oxford, UK; 3 Wellcome Trust Centre for Human Genetics, University of Oxford, Oxford, UK; 4 Craniofacial Unit, Department of Plastic and Reconstructive Surgery, Oxford University Hospitals NHS Trust, John Radcliffe Hospital, Oxford, UK; 5 Institute of Neuroscience, Newcastle University, Newcastle Upon Tyne, UK

**Keywords:** Genetics, Molecular genetics, Chromosomal, Clinical genetics, Psychiatry

## Abstract

**Background:**

Autism spectrum disorders (ASDs) are common and have a strong genetic basis, yet the cause of ∼70–80% ASDs remains unknown. By clinical cytogenetic testing, we identified a family in which two brothers had ASD, mild intellectual disability and a chromosome 22 pericentric inversion, not detected in either parent, indicating *de novo* mutation with parental germinal mosaicism. We hypothesised that the rearrangement was causative of their ASD and localised the chromosome 22 breakpoints.

**Methods:**

The rearrangement was characterised using fluorescence *in situ* hybridisation, Southern blotting, inverse PCR and dideoxy-sequencing. Open reading frames and intron/exon boundaries of the two physically disrupted genes identified, *TCF20* and *TNRC6B,* were sequenced in 342 families (260 multiplex and 82 simplex) ascertained by the International Molecular Genetic Study of Autism Consortium (IMGSAC).

**Results:**

IMGSAC family screening identified a *de novo* missense mutation of *TCF20* in a single case and significant association of a different missense mutation of *TCF20* with ASD in three further families. Through exome sequencing in another project, we independently identified a *de novo* frameshifting mutation of *TCF20* in a woman with ASD and moderate intellectual disability. We did not identify a significant association of *TNRC6B* mutations with ASD.

**Conclusions:**

*TCF20* encodes a transcriptional coregulator (also termed SPBP) that is structurally and functionally related to RAI1, the critical dosage-sensitive protein implicated in the behavioural phenotypes of the Smith–Magenis and Potocki–Lupski 17p11.2 deletion/duplication syndromes, in which ASD is frequently diagnosed. This study provides the first evidence that mutations in *TCF20* are also associated with ASD.

## Introduction

Autism spectrum disorders (ASDs) are common neurodevelopmental conditions characterised by impairments in social communication, the presence of repetitive behaviours and a restricted range of interests; intellectual disability is present in around 50% of people with ASD.^1^ ^2^ Family and twin studies show that ASDs have a strong genetic basis: at least 5–10% of siblings of children with ASD have an ASD diagnosis themselves.^2^ Siblings and parents of children with ASD are more likely than controls to show behavioural traits similar to those seen in people with ASD (the broader autism phenotype (BAP)).^3^ ^4^ Additionally, monozygotic twins are more likely to be concordant for ASD compared with dizygotic twins.^5^

Many rare mutations and variants have been shown to cause or increase the risk of ASD.^6–9^ For example, ASD occurs in several clinically defined monogenic and chromosomal disorders (including fragile X, Down, Angelman and Rett syndromes, neurofibromatosis and tuberous sclerosis). No common variants of large effect in ASD have been found^10^; however, multiple rare variants causing ASD have been identified in research and clinical settings through array comparative genomic hybridisation (CGH) and high-throughput exome and genome sequencing.^7–9^ ^11–19^ Taking account of genetic causes and other medical/neurodevelopmental conditions, the cause of ASD remains unidentified in ∼70–80% of affected individuals; hence, a substantial proportion of causative loci remains to be identified.^6–8^

The present study started with the identification of a *de novo* pericentric inversion of chromosome 22, present in two brothers who both had ASD. Further characterisation of the rearrangement revealed it to be complex, consisting of four separate chromosome 22 breakpoints physically disrupting two genes, *TCF20* (encoding transcription factor 20) and *TNRC6B* (encoding trinucleotide repeat containing 6B), both of which appeared plausible candidates for involvement in ASD. Building on this initial finding, we present additional evidence implicating *TCF20* in ASD, based both on the results of resequencing of *TCF20* and *TNRC6B* in samples from the International Molecular Genetic Study of Autism Consortium (IMGSAC) and on the separate identification of an additional *TCF20* frameshifting mutation associated with ASD. We propose that precise dosage of TCF20 is important for neurodevelopment, and that functional perturbation of TCF20 confers susceptibility to ASD.

## Materials and methods

### Patient ascertainment and diagnostic studies

Patients from two unrelated families (#1 and #6) were originally referred for assessment of coexisting craniosynostosis. Ethical approval for research into craniofacial malformations, and the specific ASD studies undertaken in these families, was provided by the Oxfordshire Research Ethics Committee B (C02.143) and the West London Research Ethics Committee (09/H0706/20), and informed consent was obtained. Genetic analyses were performed on DNA and RNA extracted from peripheral blood and lymphoblastoid cell lines. The human genome hg19 sequence release (February 2009) was used for all analyses.

### Ascertainment of ASD and control samples

Multiplex and simplex ASD families were identified, collected and assessed by the IMGSAC as previously described.^20^ ^21^ Ethical approval was obtained for the collection of all data, and written informed consent was obtained from all parents/guardians or, where appropriate, the proband. Parents were administered the Autism Diagnostic Interview-Revised (ADI-R)^22^ and the Vineland Adaptive Behavior Scales.^23^ Probands were assessed using the Autism Diagnostic Observation Schedule-Generic (ADOS-G),^24^ and a medical examination was carried out to exclude cases of known aetiology. IQ was assessed using standardised measures of verbal and performance ability.^25^ ^26^ Whenever possible, probands were karyotyped and molecular genetic testing for fragile X syndrome was performed. Family history interviews^4^ were used to investigate BAP behaviours and traits in siblings and parents when possible.

A cohort of 384 UK DNA controls from randomly selected unrelated UK Caucasian blood donors was obtained from the European Collection of Cell Cultures (ECACC) (http://www.hpacultures.org.uk/products/dna/hrcdna/hrcdna.jsp). An additional 432 locally sourced controls were tested in the case of the *TCF20* c.4670C>T variant.

### Fluorescence *in situ* hybridisation

Fluorescence *in situ* hybridisation (FISH) mapping of the chromosome 22 breakpoints in family #1 used BACs and fosmids obtained from the Children's Hospital Oakland Research Institute (CHORI); see table 1 for clone names and locations. Clones were labelled by nick-translation (Abbott Molecular) either with digoxigenin-11-dUTP (Roche) or biotin-16-dUTP (Roche). FISH was carried out following standard procedures. Briefly, the DNA probes were denatured at 75°C for 5 min and preannealed at 37°C for 45 min. Slides were denatured in 70% formamide/2× saline sodium citrate (SSC) at 70°C for 1 min and hybridised in a moist chamber at 37°C overnight. After washes in 50% formamide/1× SSC and 2× SSC at 42°C, the probes were detected with either fluorescein-conjugated antidigoxigenin (Roche) or Cy3-conjugated streptavidin (Sigma). The slides were counterstained with 4′,6-diamidino-2-phenylindole (DAPI) in Vectashield (Vector Laboratories) and analysed on a Cytovision system (Leica).

**Table 1:** Clones used for fluorescence *in situ* hybridization (FISH) analysis in family #1

Clone name	Genomic location on Chr22	Position of signal on der(22)	Breakpoint
CTA-150C2	39280232-39481326	Long arm	
WI2-1570N6	39476065-39520769	Split short/long arms	C
WI2-1013H1	39557188-39594983	Short arm	
WI2-2202O13	39516811-39555371	Short arm	
WI2-1769B14	39587327-39627092	Short arm	
WI2-3097P13	39612987-39654267	Short arm	
WI2-1881P6	39642520-39684613	Short arm	
WI2-624P20	40026816-40067597	Short arm	
WI2-1574G19	40631976-40678518	Short arm	B
WI2-1927K3	40743240-40784809	Long arm	B
CTA-250D10	42252765-42473659	Long arm	
G248P86612G1	42600994-42642421	Split short/long arms	A
RP11-241G19	42605118-42782007	Split short/long arms	A
G248P84377G7	42640176-42679204	Short arm	
RP11-794G14	43105492-43331920	Short arm	
RP11-1021O19	43972241-44158005	Short arm	
RP11-357F14	44543405-44721394	Short arm	
RP11-49A20	45141573-45322938	Short arm	
CTA-268H5	45574232-45797207	Short arm	
CTA-722E9	49795787-49928065	Short arm	
CTA-799F10	51078917-51174589	Short arm	

### Array CGH

Array CGH was performed using a human genome-wide 185K oligonucleotide array (Agilent Technologies). Genomic DNA from the inversion patient (II-4, family #1) and from a sex-matched reference were double-digested separately using the restriction endonucleases *Alu*I and *Rsa*I (Promega) and purified using Microcon centrifugal filter devices (Merck Millipore). A total of 1.5 μg of the digested products was differentially labelled by the random priming method using the fluorophores Cy3-dUTP and Cy5-dUTP (Perkin Elmer) and co-hybridised to the array for 48 h at 65°C in a rotating oven. The hybridised arrays were washed and scanned using an Agilent Microarray Scanner. The image data were extracted using Agilent Feature Extraction software V.8.5, and the data analysed using Agilent CGH Analytics software V.3.4 (*z*-score method setting).

### Single-nucleotide polymorphism array hybridisation

Genomic DNA from the inversion patient (II-4, family #1) was analysed using an ∼300K Human CytoSNP-12 BeadChip according to manufacturer's guidelines (Illumina Inc, San Diego, CA). Briefly, ∼200 ng DNA was denatured, amplified, fragmented enzymatically and hybridised to the BeadChips in an Illumina Inc. hybridisation oven at 48°C for 16–24 h. The BeadChips were washed according to the manufacturer's protocol and the hybridised DNA subjected to primer extension with labelled nucleotides prior to detection using fluorescent antibodies. Data were processed using GenomeStudioV2009.2 (Illumina Inc) and analysed using Nexus Discovery Edition v6.1 (BioDiscovery, Hawthorne, California, USA).

### Isolation of breakpoints A, B and C on chromosome 22

We obtained BACs and fosmids and performed FISH analysis, initially to identify breakpoint A (table 1). Identification of a split signal using two fosmids localised the breakpoint within ∼35 kb; single-copy probes spanning this region were synthesised and hybridised to Southern blots of patient and control DNA, further refining the breakpoint within ∼1 kb. Three breakpoint-specific primers (TSP1, 5′-GTTTTGGAGCGCCACAAAGCACT-3′; TSP2, 5′-CAAAGCACTCCCATATAAGACGGCG-3′; TSP3, 5′-AGACGGCGAACTTAATATATACATGTTGTG-3′) were combined with redundant primers in nested PCR with the DNA Walking SpeedUp Premix Kit (Seegene). After DNA sequencing to determine the site of the breakpoint and to identify the sequence and location of DNA on the other side of it (breakpoint B), a further primer pair (5′-GATAAATTTTAGCTATTATTATTACCACCTAGAAGCT-3′ and 5′-TTATAGACAAAGGCTAAGGGCAGATG-3′) was designed to confirm the breakpoint by amplifying a 1.5 kb fragment.

To identify breakpoint C, we conducted further FISH and found a split signal with BAC W12-1570N6 (table 1). We screened this ∼44.7 kb region by Southern blot analysis and identified a 15 kb *Hin*dIII fragment as likely to span the breakpoint.

### Identification of novel *TCF20* exon

A comparison of the human and mouse cDNA sequences showed that the mouse *Tcf20* transcript contains an extra exon encoding an extended 5′ untranslated region (UTR).^27^ Correspondingly, comparison of the human and mouse genomic sequence revealed a highly conserved region ∼68.5 kb telomeric of the first annotated exon of *TCF20* in the human genome. We isolated total RNA from normal human transformed B-lymphocytes and generated cDNA using random hexamer primers (RevertAid, Fermentas). Following amplification using cDNA as template with primers in the large exon of *TCF20* and the conserved region (primer pair 5′-TCCTCCCCCGCCTCGGCTCAG -3′ and 5′-CACTGCTGCCACTACTGCCACCTGTAC-3′), we found the conserved region to be spliced to the previously identified exon 1 of *TCF20,* indicating that this region represents a previously unannotated exon of human *TCF20* (GenBank KF851355).

### DNA sequencing of *TCF20* and *TNRC6B*

The entire open reading frames of *TCF20* (RefSeq accession: NM_005650.1) and *TNRC6B* (isoform 1: NM_001162501.1 and isoform 3: NM_001024843.1) were screened in the ASD panel using primers and reaction conditions shown in online supplementary table S1. Fragments were DNA sequenced on the ABI PRISM 3730 DNA sequencer, employing Big Dye Terminator mix V.3.1 (Applied Biosystems). Sequence chromatogram traces were analysed using Mutation Surveyor (Softgenetics) and Sequencher (Gene Codes). We compared the occurrence of variants in a normal control panel of 384 samples by dideoxy sequencing and examined the frequency of each variant in 8600 European American (EA) alleles from the Exome Variant Server (EVS).^28^ Synonymous and intronic variants were assessed for their potential to affect splicing using the Splice Site Prediction by Neural Network (http://www.fruitfly.org/seq_tools/splice.html), and pathogenicity of missense substitutions was investigated with PolyPhen-2 (http://genetics.bwh.harvard.edu/pph2/). Nucleotide numbering of variants in cDNA starts at the initiation ATG codon (A=1).

### Microsatellite and single-nucleotide polymorphism analysis

The haplotype surrounding the *TCF20* c.4670C>T variant identified in three families was investigated by amplifying seven flanking microsatellites (see online supplementary table S2) in proband and parental samples using primers labelled with the fluorophore 6-FAM. Fragments were analysed by capillary electrophoresis on an ABI 3730 containing POP-7 polymer, and peaks were visualised using Gene Mapper V.3.7 (Applied Biosystems). Informative single-nucleotide polymorphisms (SNPs) (see online supplementary table S2) were amplified and sequenced as described above.

Correct biological relationships of samples (and hence, exclusion of non-paternity) were confirmed in all three families with *de novo TCF20* mutations (#1, 2 and 6) using at least 10 microsatellites located on different chromosomes.

### cDNA analysis

RNA was extracted from a lymphoblastoid cell line using TRIzol/RNeasy (Qiagen) and ∼1 µg used for cDNA synthesis with random hexamers. The region containing the mutation was amplified from the proband's cDNA, an equivalent (-RT) control without addition of reverse transcriptase, and genomic DNA from proband and parents using *TCF20* Exon 2.9 primers (see online supplementary table S1), followed by a digestion with *Bsl*I and agarose gel electrophoresis.

## Results

### Chromosome 22 rearrangement associated with ASD

**Figure 1:**
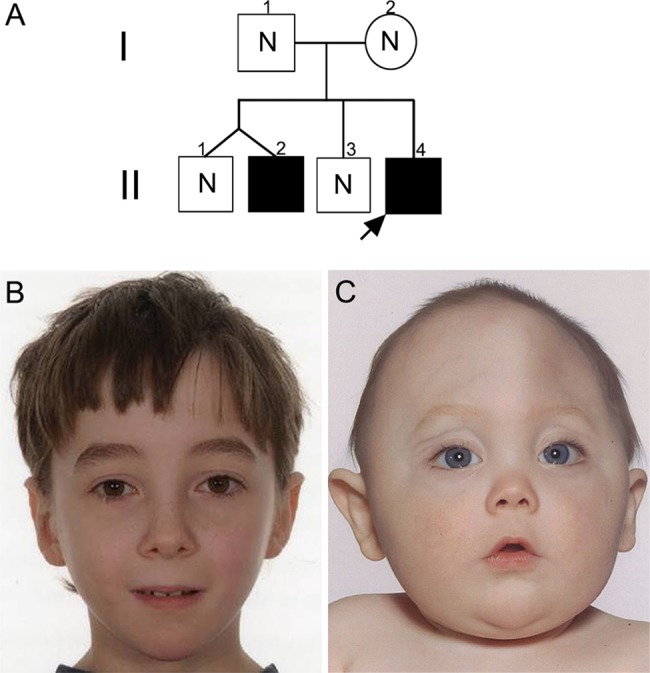
Pedigree of family #1 and facial appearance of individuals heterozygous for chromosome 22 rearrangement. (A) Pedigree showing the immediate family of the proband (arrow). Filled symbols represent individuals shown to carry the rearrangement. N indicates absence of the rearrangement. (B) Normal facial appearance of the proband's older brother II-2, aged 10 years. (C) Facial appearance of the proband aged 10 months showing trigonocephaly associated with hypotelorism and mild exorbitism, caused by premature synostosis of the metopic suture.

The proband II-4 in family #1 (pedigree, figure 1A) was assessed at the age of 7 months because of an abnormal craniofacial appearance (figure 1C). Plain radiographs and CT of the skull showed fusion of the metopic and coronal sutures and extensive copperbeating suggestive of raised intracranial pressure; the brain appeared structurally normal. Subtotal calvarial remodelling was performed at the age of 1 year. Karyotyping of peripheral lymphocytes revealed a pericentric inversion of chromosome 22, reported as 46,XY,inv(22)(p11?.2-q13?.1). Testing of the family showed the same abnormal karyotype in his older brother, who had no craniofacial dysmorphism (II-2; figure 1B); surprisingly, the karyotypes of both parents (I-1 and I-2), as well as the other two siblings (II-1 and II-3), were normal. During childhood, the two brothers with the inversion (II-2, II-4), but not their siblings or parents, were diagnosed with clinical autism and mild intellectual disability by their local clinicians; subsequently, both individuals met autism criteria during research assessments using ADOS-G (table 2). Array CGH of DNA from the proband was performed using 185K and 300K genome-wide oligonucleotide arrays (see ‘Methods’), but neither revealed any significant gain or loss of material.

**Table 2:** Summary results of Autism Diagnostic Observation Schedule-Generic (ADOS-G) and IQ/developmental assessments in subjects with *TCF20* mutations

Family #	Patient ID	TCF20 abnormality	ADOS-G social communication score (age at assessment in years)	IQ/developmental quotient (test, age at assessment in years)
1	II-4 (proband)	Inversion break intron 1	13 (10 years)	Full scale 79, verbal 79, performance 79 (WPPSI-3, 3.5 years)
1	II-2 (brother)	Inversion break intron 1	16 (12 years)	Communication 45, daily living 55, socialisation 44 (VABS, 7 years)
2	proband	p.K512E	16 (7 years)	Full scale 120 (WASI, 13 years)
3	proband	p.P1557L	11 (8 years)	Performance 100 (Raven's matrices)
4	proband	p.P1557L	NA	NA
5	proband	p.P1557L	11 (10 years)	Performance 80 (Raven's matrices)
5	brother	p.P1557L	NA	Performance 107 (Raven's matrices)
6	proband	p.K1173Rfs*5	12 (25 years)	Full scale 45, verbal 50, performance 47 (WISC-3, 14 years)

NA, not available; VABS, Vineland Adaptive behaviour Scales; WASI, Wechsler Abbreviated Scale of Intelligence; WISC, Wechsler Intelligence Scale for Children; WPPSI, Wechsler Preschool and Primary Scale of Intelligence.

**Figure 2:**
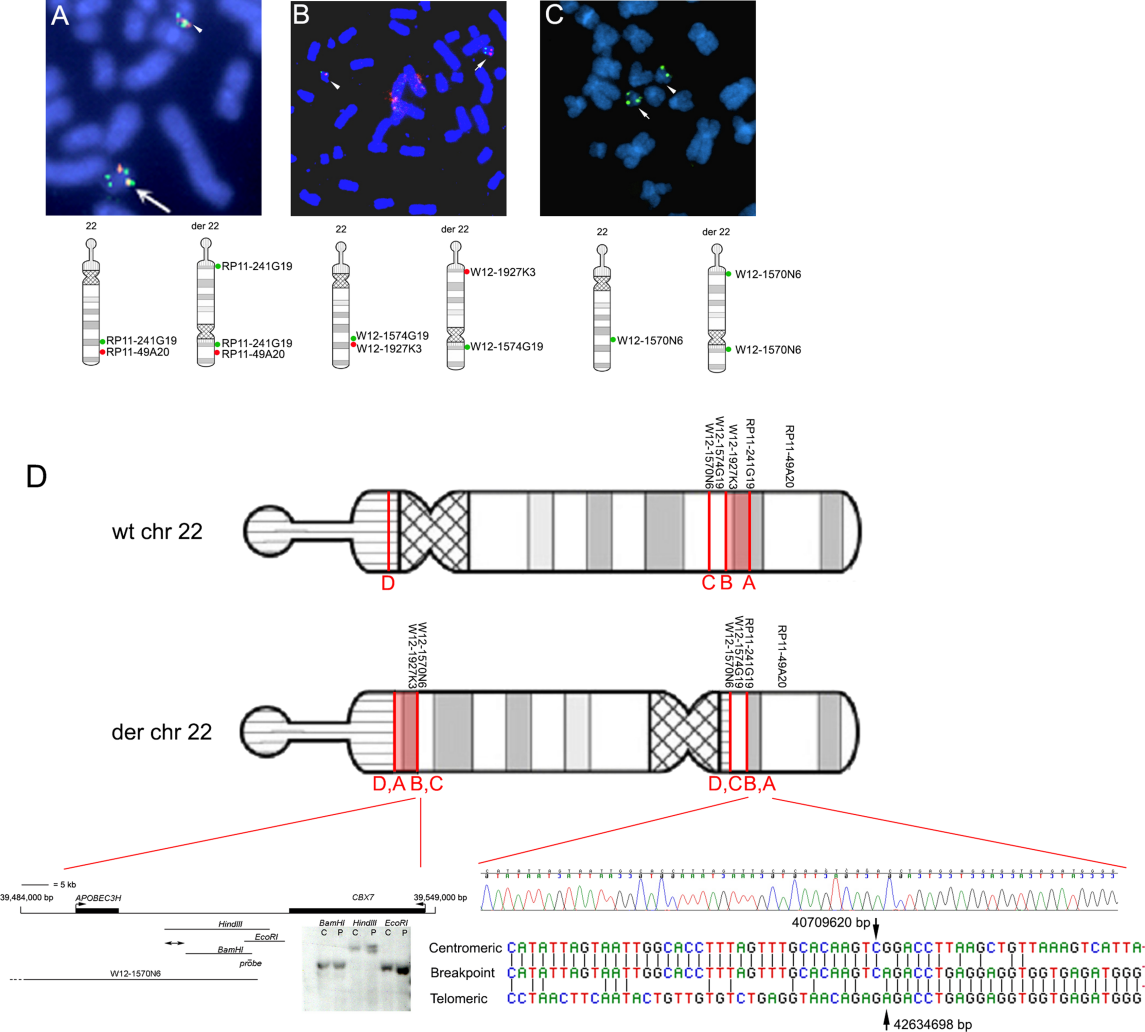
Structure of the chromosome 22 rearrangement deduced from fluorescence *in situ* hybridization (FISH) analysis and DNA sequencing. (A–C) Representative FISH analysis and diagrammatic interpretation of structure of the rearranged chromosome (der22), shown in more detail with positions of breakpoints in (D). (A) Signals from RP11-241G19 (green), which spans breakpoint A, and the more distal RP11-49A20 (red) are adjacent on the normal chromosome 22 (arrowhead) but a split green signal is seen near the opposite end of the der22 (arrow). (B) Clones W12-1927K3 (red) and W12-1574G19 (green), which lie on either side of breakpoint B, showing hybridisation together on the normal chromosome 22 (arrowhead) and at opposite ends of the der22 (arrow). C. Single signal with W12-1570N6 on normal chromosome 22 (arrowhead), but split signal on derived 22 (arrow) indicating position of breakpoint C. (D) Ideograms of wt and derived chromosome 22. The order of BAC and fosmid clones employed in figure parts A–C is shown, together with the locations of breakpoints A–C. The 2 Mb region between breakpoints A and B is shown in light red (orientation on the derived chromosome is uncertain). Breakpoint D on the satellite short arm was not further characterised. Below left, map of the 65 kb region that includes breakpoint C, showing the positions and orientations of genes. The Southern blot analysis shows an apparent breakpoint in the patient sample (P) compared with the control (C), localising the breakpoint to the indicated segment (double-ended arrows) of ∼4 kb. Below right, the DNA sequence chromatogram spanning the breakpoints A and B is shown above an alignment of this sequence with the normal sequences at the telomeric and centromeric ends of breakpoints. Arrows indicate positions and numbering of the last intact bases on either side of the translocated region.

To characterise the molecular nature of the pericentric inversion, we performed FISH using multiple BACs and fosmids (table 1). These probes were initially focused on the 22q13.1 band in which the long-arm breakpoint had been tentatively located, but several further rounds of analysis were performed as greater complexity in the rearrangement became apparent (figure 2). The observation of split signals with two fosmids localised one breakpoint (termed breakpoint A) to a ∼37 kb region (figure 2A). Further analysis by Southern blotting with single-copy probes identified breakpoint fragments, initially within a ∼15 kb *Eco*RI fragment, and subsequently within a 248 bp fragment bordered by *Stu*I and *Afl*III restriction sites (not shown). PCR primers were designed to amplify across the breakpoint in sequentially nested amplifications with degenerate primers (see ‘Methods’). Surprisingly, DNA sequencing of this amplification product identified the sequence on the centromeric side of the break as originating from a location ∼1.9 Mb centromeric of breakpoint A (figure 2D, bottom right). These sequence data showed contiguity between nucleotides at coordinates at 40 709 620 bp (breakpoint B) and 42 634 698 bp (breakpoint A), adjacent to a short stretch of 5-nucleotide (5′-GACCT-3′) complementarity (figure 2D). Confirming the identification of breakpoint B, clones closely adjacent on either side of this location mapped to opposite arms of the der(22) (figure 2B). This result implied that a third more centromeric break on the long arm (‘breakpoint C’) must have occurred, to which the intermediate segment (B-A) had been joined. This break was localised using FISH to an ∼44.7 kb region within BAC clone W12-1570N6 (figure 2C). Analysis by Southern blotting revealed a *Hin*dIII restriction fragment that likely spanned the breakpoint (figure 2D, bottom left), locating the breakpoint to a ∼4 kb region between 39 507 139  and 39 511 083 bp. Figure 2D summarises the structure of the derivative chromosome 22 as concluded from the FISH, Southern blotting and DNA sequencing results. Breakpoint D is predicted to occur in the short arm satellite sequence of chromosome 22 and was not characterised further. Although (as demonstrated by array CGH) there has been no major gain or loss of material at the breakpoints, we found evidence of a small (∼10 kb) duplication at breakpoint A (data not shown) and this may apply to others too, most consistent with the replication-based fork stalling template switching (FoSTeS)-type mechanism for the complex chromosome rearrangement.^29^

### Gene content at breakpoints A, B and C and selection of *TCF20* and *TNRC6B* for further analysis

**Figure 3:**
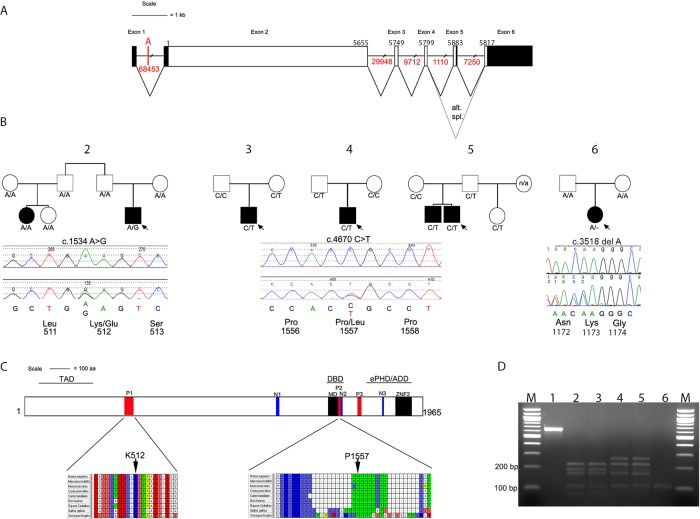
*TCF20* gene structure, identification of variants in ASD cases and their location within conserved domains. (A) Schematic representation of *TCF20*, exons are shown to scale with the coding sequence in white and untranslated regions filled in with black. There is an alternative stop codon in the alternatively spliced exon 5. The position of the first coding nucleotide is shown in exon 2, numbers above boxes indicate cDNA numbering at last nucleotides of exon boundaries or last nucleotide of stop codons; numbers in red below lines indicate intron sizes (not to scale). The location of breakpoint A that interrupts *TCF20* 23350 bp 5′ of exon 2 is also indicated. (B) Pedigrees of five families with variants of *TCF20* that are either novel or enriched compared with control samples. Below each pedigree is a chromatogram showing the sequence change together with the amino acids encoded by the change and by adjacent codons. Black symbols indicate individuals with a clinical and research ASD diagnosis, the white symbol indicates people without clinical ASD; where broader autism phenotype data are available this is described in the text; n/a indicates that no DNA was available for analysis. Under each symbol, the status of that individual for the change found in the proband is shown. (C) Diagram representing the TCF20 protein with previously annotated domains: P1-P3, PEST domains; N1-N3, nuclear localisation signals; MD, minimal DNA binding domain; ZNF2, zinc finger domain. The three lines above the protein denote the following domains: TAD, transactivation domain; DBD, DNA binding domain and the ePHD/ADD domain.^37^ The lower panel shows the positions and conservation of amino acid residues predicted to be substituted in ASD pedigrees. The entire PEST1 and PEST2 sequences are shown with interspecies conservation in mammals, chicken and frog. (D) Analysis of cDNA amplification product compared with genomic (gDNA) from region containing c.3518delA mutation in family #6. Restriction digestion was performed with *Bsl*I, yielding product sizes (bp) of 215, 162, 145, 72, 1 in the absence of the mutation and 233, 215, 145, 1 in the presence of the mutation. Lanes numbered as follows: 1, undigested gDNA from proband; 2, mother's gDNA; 3, father's gDNA; 4, proband's gDNA; 5, proband's cDNA and 6, −RT control for proband's cDNA. Note similar relative intensities of mutant and non-mutant fragments in lanes 4 and 5, indicating lack of significant nonsense-mediated decay associated with the frameshifting mutation.

We analysed the three breakpoints on the long arm of chromosome 22 to determine whether they disrupted any genes. Initially breakpoint A appeared to locate within an intergenic region; however, because of sequence homology with the mouse orthologue of *TCF20* in which an extra exon is described,^27^ we predicted the existence of a previously unannotated exon located 5′ of the currently annotated first exon of human *TCF20*. Primers for cDNA analysis of the corresponding human region were designed (see ‘Methods’); starting with RNA isolated from transformed B lymphocytes, we found this region is indeed spliced to the previously described first exon of *TCF20* (see online supplementary figure S1). This novel exon of the human *TCF20* transcript encodes an extended 5′ UTR. Therefore, breakpoint A disrupts *TCF20* in intron 1 at a position 23.3 kb 5′ of exon 2 (figure 3A). *TCF20* encodes a transcriptional coregulator paralogous to *RAI1*, the causative gene in Potocki–Lupski syndrome (duplication of 17p11.2), which is associated with ASD in ∼90% of cases;^30^ ^31^ deletions of this region cause Smith–Magenis syndrome, characterised by severe intellectual disability and neurobehavioural problems, including ASD.^32^ ^33^ Breakpoint B locates within intron 19 of *TNRC6B*, which encodes a product that stably associates with argonaute proteins required for microRNA-guided mRNA cleavage.^34^ Breakpoint C does not apparently disrupt any genes, occurring >12 kb telomeric of *APOBEC3H* and >5 kb centromeric of *CBX7* (figure 2D, bottom left).

We hypothesised that the ASD present in the two brothers with the complex chromosome 22 rearrangement was most likely due to altered function of one or both of the two physically disrupted genes, *TCF20* and *TNRC6B*. There is no established abnormal phenotype associated with mutations in either of these two genes, or in their murine orthologues, although there are reports of copy number variations (CNVs) that include *TNRC6B* being linked to ASD (see ‘Discussion’). We therefore proceeded to resequence both genes in the large number of families recruited by IMGSAC.

### Resequencing of *TCF20* and *TNRC6B* in the IMGSAC cohort

*TCF20* comprises six exons, five of which encode two open reading frames of 5880 and 5814 nucleotides generated by alternative splicing (figure 3A). *TNRC6B* is alternatively spliced to generate multiple isoforms, including 25 different coding exons. We undertook DNA sequencing of the coding sequences of both genes, including the intron–exon boundaries, in 342 unrelated ASD probands (260 from multiplex pedigrees and 82 from simplex families) from the IMGSAC cohort, and where possible performed parent and sibling studies of the rare variants identified. The occurrence of all variants likely to be functionally relevant (either amino acid altering or predicted to affect splicing) was compared with normal control data as described in ‘Methods’. The results for *TCF20* are summarised in table 3 and those for *TNRC6B* in online supplementary table S3.

**Table 3:** Amino acid sequence altering variants of *TCF20* found in 342 ASD samples, comparison with controls, and family follow-up

Nucleotide change	Amino acid change	Number of heterozygous ASD samples/total sequenced†	Number of heterozygous control samples/total sequenced†	Exome Variant Server (EA) expressed as rare/common alleles	Family follow-up	PolyPhen-2 prediction
c.47G>C	p.S16T	10/331	8/353	123/8477	–	Benign (0.015)
c.162_167del	p.S55_G56del	2/331	3/353	35/8219	–	n/a
c.del966_968	p.Q322del	1/336	2/354	11/8243	–	n/a
c.1213A>G	p.M405V	63/338 [4]	61/351 [3]	788/7812	–	Benign (0)
c.1534A>G	p.K512E	1/337	0/352	0	*De novo*	Probably damaging (0.970)
c.2164A>G	p.S722G	102/338 [19]	119/354 [8]	1797/6803	–	Benign (0)
c.3495G>A	p.M1165I	1/335	0/356	11/8589	–	Benign (0.01)
c.4670C>T	p.P1557L	3/335	0/793	3/8597	See figure 3	Probably damaging (0.963)
c.5810C>T	p.P1937L	1/339	0/354	2/8598	Absent in affected sibling; present in unaffected sibling	Probably damaging (0.988)
c.5825C>A	p.P1942H	1/339	0/354	1/8599	Absent in affected half-sibling; transmitted by non-shared parent	Possibly damaging (0.634)

†The number of samples from each panel found to harbour the variant is shown next to the number of samples successfully screened. Numbers in square brackets refer to homozygous changes.

EA, European American.

In *TCF20,* we identified two common SNPs and eight different rare heterozygous changes (encoding two in-frame deletions and six non-synonymous substitutions), each present in between 1 and 10 ASD probands. Common SNPs did not differ in frequency between cases and controls. Of the rare variants, six were considered unlikely to be causally contributory either because they were present at significant frequency in the EVS (n=4) or an affected sibling did not inherit the variant allele (n=2). The remaining two variants (c.1534A>G and c.4670C>T), identified in one and three different families respectively, were considered potentially pathogenic. The dideoxy sequencing and segregation of these variants is shown in figure 3B, and the positions of the encoded missense changes in the TCF20 protein domain structure and species conservation in figure 3C. In the multiplex ASD family #2, the c.1534A>G transition encodes a likely damaging p.K512E substitution (PolyPhen-2 score 0.97), which had arisen *de novo* in the proband. This individual had classical Asperger syndrome with good intellectual function (table 2), whereas his cousin had autism, severe intellectual disability and early-onset epilepsy; phenotyping with the family history interview revealed evidence of the BAP in two other family members. Correct biological relationships were confirmed by microsatellite analysis and by haplotype analysis based on a 1M SNP chip (data not shown). The variant was absent in 352 ECACC samples experimentally tested, and not recorded in 6503 samples from the EVS. Amino acid position 512 locates within the PEST1 sequence of TCF20 and is highly conserved in vertebrates (figure 3C); PEST sequences provide targets for proteolytic protein degradation.^35^

In unrelated ASD probands from three families (singleton families #3 and #4 and multiplex family #5), a c.4670C>T transition encoding p.P1557L (PolyPhen-2 score 0.963) was present (figure 3B). Proline 1557 locates within the PEST2 domain of TCF20 and is highly conserved in vertebrates (figure 3C). The c.4670C>T variant was inherited from the mother (about whom there are no phenotypic data) in family #3 and from the father in families #4 and #5. In family #5, both boys had ASD and average range IQ; the father had evidence of the BAP. The frequency of this substitution in the ASD cohort (3/342 individuals) is significantly higher (Fisher's exact test) than in control populations, based both on our own resequencing data (0/793; p=0.027) and from EVS (3/4,300; p=0.007). Observing that this C>T transition has arisen at a hypermutable CpG site, we analysed the haplotype background on which each variant T allele was present. Using microsatellites and SNPs within a 0.54 Mb region around the substitution that contains no recombination hotspots (defined as ≥10 cM/Mb) according to the International HapMap Consortium (http://hapmap.ncbi.nlm.nih.gov/), we found multiple differences between each of the three haplotypes (table 4), including different alleles in family #5 for SNPs (rs16986035 and rs1548304) that flank the c.4670C>T variant. These data are consistent with the mutation having arisen independently at least twice.

**Table 4:** Microsatellite and single-nucleotide polymorphism (SNP) markers in ∼0.5 Mb region surrounding *TCF20* to distinguish c.4670C>T haplotypes in families 3, 4 and 5

	Tcms4* 42390888 bp	Tcfms3 42433133 bp	Tcfms2 42433855 bp	Tcfms1 42544517 bp	rs2899354 42554409 bp	rs4453786 42563308 bp	rs16986035 42602139 bp	c.4670C>T 42606642 bp	rs5758652 42612408 bp	rs1548304 42691488 bp	rs6002674 42694220 bp	rs11704558 42695148 bp	rs6002676 42697216 bp	Tcfms5 42775494 bp	Tcfms6 42782403 bp	Tcfms7 42939056 bp	Tcfms8 43053571 bp
Family 3 (father)	158/162	200/202	203/203	200/214	C/A	A/G	A/A	C/C	A/G	C/T	C/T	C/C	G/A	182/184	164/166	202/202	219/223
Family 3 (mother)	156/156	206/206	205/205	204/206	C/C	G/G	G/G	C/T	A/A	C/C	T/T	C/T	G/A	184/184	166/166	202/206	213/223
Family 3 (proband)	156/162	202/206	203/205	200/206	C/C	G/G	G/A	C/T	A/A	C/C	T/T	C/C	A/A	182/184	164/166	202/206	213/223
**Family 3 c.4670T haplotype†**	**156**	**206**	**205**	**206**	**C**	**G**	**G**	**T**	**A**	**C**	**T**	**C**	**A**	**184**	**166**	**206**	**213**
Family 4 (father)	156/162	200/206	201/205	204/214	C/A	A/G	G/A	C/T	A/G	C/T	T/T	C/C	A/A	184/186	166/166	202/202	219/225
Family 4 (mother)	156/162	200/206	203/205	200/204	C/C	G/G	A/G	C/C	A/A	C/C	T/T	T/T	G/G	188/188	164/166	202/202	219/221
Family 4 (proband)	162/162	200/206	203/205	200/204	C/C	G/G	G/G	C/T	A/A	C/C	T/T	C/T	G/A	186/188	166/166	202/202	219/221
**Family 4 c.4670T haplotype**	**162**	**200/206**	**205**	**204**	**C**	**G**	**G**	**T**	**A**	**C**	**T**	**C**	**A**	**186**	**166**	**202**	**219**
Family 5 (mother)	162/162	200/202	201/205	200/200	C/C	G/G	G/G	C/C	A/A	T/C	C/T	C/T	G/G	184/186	166/170	202/202	219/223
Family 5 (father)	156/162	200/206	203/205	200/204	C/A	G/A	A/G	C/T	A/G	T/T	T/T	C/C	G/A	186/186	166/168	202/202	219/221
Family 5 (proband)	162/162	200/206	203/205	200/204	C/C	G/G	A/G	C/T	A/A	T/C	T/T	C/T	G/A	186/186	166/170	202/202	219/219
**Family 5 c.4670T haplotype**	**162**	**206**	**203**	**204**	**C**	**G**	**A**	**T**	**A**	**T**	**T**	**C**	**A**	**186**	**166**	**202**	**219**

*See online supplementary table S2 for details of markers.

†The haplotype associated with the c.4670C>T change in each family is shown in bold, markers in each pedigree that differ from those in the other two pedigrees are underlined.

In the case of *TNRC6B*, we identified 12 different rare non-synonymous changes (encoding 1 frameshift, 1 single amino acid deletion and 10 missense substitutions) each present in 1 or 2 of 335 IMGSAC samples sequenced (see online supplementary table S3). Of these, six were deemed unlikely to be causally contributory because they were previously identified at significant frequency in the EVS (n=4), the mutation was predicted as being functionally benign (n=1) or an affected sibling did not inherit the variant allele (n=1). In the remaining six cases (which include the frameshift and the amino acid deletion), and in contrast to *TCF20*, none was shown to have arisen *de novo* or to show a significant frequency difference between cases and controls (although lack of availability of DNA samples from some family members prevented complete analysis). Hence, these data are inconclusive regarding a contribution of *TNRC6B* mutations to ASD in the IMGSAC cohort.

### A *de novo* truncating mutation of *TCF20* in an individual with ASD and intellectual disability

While this work was being undertaken, we coincidentally discovered a further *TCF20* mutation during an unrelated project aimed at identifying novel genetic causes of craniosynostosis.^36^ The exome sequence from a woman with unicoronal synostosis segregating from her mother (family #6) was found to contain a heterozygous one-nucleotide deletion of *TCF20* (c.3518delA encoding p.K1173Rfs*5). Analysis of parental samples showed that it had arisen *de novo*, indicating that it was not causative of the familial craniosynostosis (figure 3D). Clinical case note review revealed that the proband had clinically diagnosed ASD and moderate intellectual disability; she subsequently met autism criteria during a research ADOS (table 2).

To determine whether this *TCF20* mutation causes nonsense-mediated mRNA decay, we analysed cDNA obtained from a lymphoblastoid cell line from the proband. Unexpectedly, this showed equal representation of the normal and mutant alleles in the cDNA product (figure 3D), indicating that the mutant mRNA is stable; hence, a truncated protein is expected to be produced in significant quantities.

## Discussion

Starting with the clinical observation of the concurrence of a *de novo* chromosome 22 inversion and ASD phenotype in two male siblings, we have accumulated three lines of evidence supporting a causative association between disruption of *TCF20* and ASD, which was not identified by recent exome or genome sequencing studies.^14–19^ First, the original inversion separates the coding portion of *TCF20* from a previously unannotated upstream untranslated exon that is conserved in mice, and therefore likely to have an important function. Second, we identified two *de novo* mutations of *TCF20* (one encoding a missense change in a predicted PEST domain, the other a one-nucleotide deletional frameshift) in individuals with ASD. Third, we identified a significant association of ASD with a likely recurrent missense variant in a second predicted PEST domain of TCF20. Although we do not exclude a contributory role for disruption of *TNRC6B* to the ASD phenotype in family #1 (indeed, single CNV-based deletion and duplication events in ASD cases that include *TNRC6B* were previously catalogued),^12^ ^13^ ^16^ the evidence from our own study is more compelling for the contribution of *TCF20*, which is the focus of this discussion.

*TCF20* (also termed SPBP, SPRE-binding protein) encodes a transcriptional coregulator,^37^ initially identified by its ability to bind the stromelysin-1 PDGF-responsive element (SPRE) element of the stromelysin-1 (matrix metalloproteinase-3/MMP3) promoter.^38^ Although widely expressed, *TCF20* shows notably increased expression in premigratory neural crest cells^39^ and in the developing mouse brain at E13.5,^40^ with specific enrichment in the hippocampus and cerebellum.^41^ This brain expression pattern is consistent with a role in ASD.^42^ Significantly, TCF20 contains seven regions with 97% sequence similarity to RAI1,^27^ mutations and deletions of which underlie Smith–Magenis syndrome.^32^ The two proteins show an overall 45% similarity and share organisation of several domains such as the three nuclear localisation signals, a C-terminal extended PHD domain and an N-terminal transactivation domain.^37^ ^43^ There is also striking similarity in the gene structure of *RAI1* and *TCF20*, with over 90% of the coding region of each located in one exon that also contains the start codon, suggesting that *TCF20* and *RAI1* evolved from a common ancestor by genome duplication.^43^ A yeast two-hybrid screen with the ZNF2 domain of TCF20 as bait identified RAI-1 as a binding partner, showing that these proteins are able to interact and therefore may also be functionally related.^27^

To test whether mutations of *TCF20* play a wider role in ASDs, we screened the coding sequence in 342 IMGSAC samples. We found two missense mutations of likely functional significance (see figure 3). One of these, p.K512E, had arisen *de novo* in the proband. Given that a total of 2 018 826 bp were screened in the ASD samples (5903 bp *TCF20* coding region in 342 samples) and assuming a germline mutation rate of 1.2×10^−8^,^44^ the chance of coincidentally identifying an unrelated *de novo* variant is ∼0.05. Hence, the *de novo* nature of the p.K512E mutation favours a causal contribution to ASD, and the high evolutionary conservation of the K512 residue is consistent with this (figure 3C). Of note, a cousin of the proband also had ASD but did not carry the variant (figure 3B), suggesting that there is genetic heterogeneity for ASD causation within this family.

The second *TCF20* variant of note is the c.4670C>T (p.P1557L) substitution identified in three ASD individuals from 335 successfully screened for this amplicon. In each case, the variant was present in one of the parents without ASD; no BAP data were available for two parents. In the multiplex family (#5), it was inherited from a father with evidence of BAP and also present in both an affected brother and a half-sister without ASD but for whom no BAP data were available. Haplotype analysis of the individuals carrying the c.4670C>T transition in the three families strongly supports an independent origin in family 5 compared with the other two families (table 4), compatible with the notion that the p.P1557L substitution confers selective disadvantage but is maintained at a low level in the population by recurrent mutation at the CpG dinucleotide.

Of note, the two ASD-associated missense changes in *TCF20* each locates within a different PEST domain. PEST sequences are so-called because of enrichment in proline (P), glutamic acid (E), serine (S) and threonine (T) and are common in proteins that are rapidly degraded in eukaryotic cells^35^ and interact with Cul3, a subunit of a Cullin-RING ubiquitin E3 ligase complex that polyubiquitinates proteins.^45^ Loss of PEST motifs occurs, for example, in NOTCH1 and NOTCH2 mutations that characterise T-cell acute lymphoblastic leukaemia^46^ and Hajdu–Cheney syndrome,^47^ respectively. Hence, these observations suggest that the ASD-associated mutations might stabilise the protein rather than causing a haploinsufficiency. Alternatively, the p.P1557L substitution might affect the nucleosome-binding activity associated with this region of the TCF20 protein.^43^

The final piece of evidence linking *TCF20* with ASD came serendipitously, while studying the genomic origins of craniosynostosis in an unrelated study. Exome sequencing of family #6 revealed a heterozygous mononucleotide frameshifting mutation of *TCF20* in a woman with craniosynostosis, a phenotype that was also present in her mother. Given that the mother was of at least average range IQ, the ASD and moderate intellectual disability in her daughter were unexpected and were not thought to be directly related to the coincident craniosynostosis. In the context of our other findings, the *de novo TCF20* mutation now provides a plausible explanation for the proband's phenotype. Although it might be expected that this mutation would lead to haploinsufficiency, cDNA analysis showed that the mutant message is stable (figure 3D). The more C-terminal PEST domain would be absent in the translated product, which could, as in the case of the missense mutations, stabilise the protein against degradation.

In summary, we propose that *TCF20* mutations constitute a newly identified contributor to ASD that was not highlighted by recent genome-wide screens.^12–19^ *TCF20* mutations may also contribute to intellectual disability, although not all individuals with mutations had his phenotype (table 2). Interestingly none of the mutations presented here predicts simple haploinsufficiency; this may explain why deletions of *TCF20* have not been observed in previous extensive CNV screens of ASD. Rather, the pathophysiological mechanism may involve the persistence or misexpression of *TCF20* in critical tissues or timepoints: this possibility should be addressed in future functional studies. Of particular interest in this regard, both underdose and overdose of the paralogous RAI1 protein cause overlapping neurological symptoms, suggesting that *RAI1* gene dosage is critical in specific neurodevelopmental pathways.^48^ Given the likely functional overlap between *TCF20* and *RAI1*, our observations provide strong support for further investigation of the normal functions of TCF20 in neurodevelopment and the role of mutations in ASD.

A recent meta-analysis of genome-wide association studies in schizophrenia^49^ identified a significant association with a SNP (rs6002655) lying within an intron of *TCF20*. This raises the possibility that variation in TCF20/SPBP function may impact neuropsychiatric disorders additional to ASD.

## Supplementary Material

Web supplement
