# Supplementary material for: De novo and rare inherited mutations implicate the transcriptional coregulator TCF20/SPBP in autism spectrum disorder
Source: J Med Genet. 2014 Sep 16;51(11):737–47. doi: 10.1136/jmedgenet-2014-102582 (PMC4215269; doi:10.1136/jmedgenet-2014-102582)
Supplement: Web supplement [file jmedgenet-2014-102582-s1.pdf]

Additional online material

***De novo* and rare inherited mutations implicate the transcriptional coregulator TCF20/SPBP in autism spectrum disorder** by Christian Babbs, Deborah Lloyd *et al*

Supplementary note

Members of International Molecular Genetic Study of Autism Consortium (IMGSAC)

UK

Jeremy R. Parr, Institute of Neuroscience, Newcastle University, UK; Jeremy.Parr@ncl.ac.uk
A. S. Le-Couteur, Institute of Health and Society, Newcastle University, UK; a.s.le-couteur@ncl.ac.uk
Gillian Baird, Guy's and St Thomas' NHS Trust & King's College London, UK; gillian.baird@gstt.nhs.uk
Andrew Pickles, Institute of Psychiatry, King's College London, UK; andrew.pickles@kcl.ac.uk
Patrick Bolton, Institute of Psychiatry, King's College London, UK; patrick.bolton@kcl.ac.uk
Alistair Pagnamenta, Wellcome Trust Centre for Human Genetics, University of Oxford, UK; alistair@well.ox.ac.uk
Richard Holt, Wellcome Trust Centre for Human Genetics, University of Oxford, UK; richard.holt@ndcn.ox.ac.uk
Inês Girão Meireles de Sousa, Wellcome Trust Centre for Human Genetics, University of Oxford, UK; ines.meireles.sousa@gmail.com
Nuala Simpson, Wellcome Trust Centre for Human Genetics, University of Oxford, UK; nuala.simpson@well.ox.ac.uk
Simon Wallace, University of Oxford Department of Psychiatry, UK; Simon.Wallace@autistica.org.uk
Kerstin Wittemeyer, School of Education, University of Birmingham, UK; k.wittemeyer@bham.ac.uk
Jonathan Green, Academic Department of Child Psychiatry, University of Manchester, Manchester, UK; jonathan.green@manchester.ac.uk
Janine Lamb, Centre for Integrated Genomic Medical Research, The University of Manchester, Manchester, UK; Janine.Lamb@manchester.ac.uk

EU

Sabine Klauck, Division of Molecular Genome Analysis, German Cancer Research Center (DKFZ), Heidelberg, Germany; s.klauck@dkfz-heidelberg.de
Fritz Poutska, Department of Child and Adolescent Psychiatry, Psychosomatics and Psychotherapy, J.W. Goethe University Frankfurt, Germany; poustka@em.uni-frankfurt.de
Maretha de Jonge, Department of Child Psychiatry, Utrecht, The Netherlands; M.V.Jonge@umcutrecht.nl
Herman van Engeland, Department of Child Psychiatry, Utrecht, The Netherlands; H.vanengeland@planet.nl
Bernadette Roge, Centre d'Etudes et de Recherches en Psychopathologie, Toulouse, France; roge@univ-tlse2.fr
Sven Bolte, Department of Women's and Children's Health, Karolinska Institutet, Stockholm, Sweden; sven.bolte@ki.se
Lennart Pedersen, Center for Autisme, Denmark; lp@centerforautisme.dk
Torban Isager, University Centre for Child and Adolescent Psychiatry Vibeholmsvej, Glostrup, Denmark; torben.isager@dadlnet.dk

Elena Maestrini, Dipartimento di Farmacia e BioTecnologie, Università di Bologna; elena.maestrini@unibo.it
Elena Baccelli, Dipartimento di Farmacia e BioTecnologie, Università di Bologna; elena.bacchelli@unibo.it
John Tsiantis, University Department of Child Psychiatry, Athens, Greece; itsianti@cc.uoa.gr

Canada
Anthony Bailey, Department of Psychiatry, University of British Columbia, Vancouver, Canada; anthony.bailey@ubc.ca

USA
Anthony Monaco, Tufts University, Boston, Massachusetts, USA; Anthony.Monaco@tufts.edu
Edwin Cook, Institute for Juvenile Research, Department of Psychiatry, University of Illinois at Chicago, Chicago, USA; ecook@psych.uic.edu
Steven Guter, Department of Psychiatry, University of Illinois, Chicago, Illinois, USA; sguter@psych.uic.edu
Fred Volkmar, Child Study Centre, Yale University, New Haven, Connecticut, USA; fred.volkmar@yale.edu
Catherine Lord, Weill Cornell Medical College/ New York Presbyterian Hospital, Columbia University, White Plains, NY, USA; cal2028@med.cornell.edu
Bennett Leventhal, Nathan Kline Institute for Psychiatric Research (NKI), Orangeberg, NY, USA; Bennett.Leventhal@NKI.RFMH.org
Eric Fombonne, Oregon Health & Science University, Institute for Development & Disability, Department of Psychiatry, Portland, USA; fombonne@ohsu.edu

Table S1. Primers used to amplify[a] and sequence *TCF20* and *TNRC6B*

Amplicon	Forward Primer 5'-3'	Reverse Primer 5'-3'
TCF20		
Exon 2.1	GGCAGAAGTTGCATTGAGCTGAGATCGTG	ATCGCTGCCGCAGCAGCTGCTGCTC
Exon 2.2	CCGGCTAGAAGAGTTCAGCCCTCGTCAG	GAGGATGGCAGAGTTGAGGGCCGCTGCAT
Exon 2.3	TTACCAGTCCCATCAGCCCCTGCCACAG	ACCTCACAGGAACCTCAGGCTGGTTGTACTG
Exon 2.4	GCAACAACAACACCCTTCTCAGCATGTGATGCAGT	GAATTTGTGCAGCTATCTGCTTTCTTGGAAGATGAG
Exon 2.5	GGCCAATCTTCCTAACACTGTCCAGCACATG	TAGGTGGCCTTTGAGTGGCTGCAGGATCATC
Exon 2.6	GGTGATTGTCTCCCGGGAAGCCATGACAG	GCCATCCCCTGATGCTCTTGAGTACTCCTAG
Exon 2.7	TTCCCAAGCCTCCTGCAGGAAGTGCTTCAG	CATTCCTCCCAATTCTGGTGTCGGCACTCATG
Exon 2.8	CTGTGATATTTCTCCACTAAGACAGATTGTCAG	TGATAAGCAGAGGCCAGGGTTTCTGAGT
Exon 2.9	GGAGACCCTCATCACATGAATCCACACATGAC	CCTCAGCTAGTCCATGTCCATCAGTCTCATG
Exon 2.10	GCAAACTTCTCCAGCCAAAAGCAGCGGTCCT	GAGGGTAACAGCAGGGCAGTTTCTACTATCTG
Exon 2.11	CAATTCCTATGCCCATCTTTCTCACAGTCAG	AACTGTTTCTGCATGTGTCTCTGTCTTCACT
Exon 2.12	CAGTCCAGCAAACCAGGAGTTGCACGTAG	GTGGCTGGGTGGCATATTTTAGTTTGATCTCAG
Exon 2.13	CCCCACAGATACCAGAAGGTTCTGCAGATG	AGGTGGATTCTTCGGGAGAGTGGCTGCA
Exon 2.14	GCCAGTTACCGGAACATGGGTGAC	GGCACTTTAATTTGGAGGAATAATCATGTGACATG
Exon 3	GAG ACA CGT GCT GAG GTC TAA CAG ATG	CTGTATGTCCTAATCCTTTGGATGCTCTG
Exon 4	CAA TCC CAG CCA GCA TGA CCA CAC CTA TGT C	TTCAGGAGGAGCCACCCTCGATCCCATC
Exon 5	CCT GAT TTC TGC ACT GTC CTC TCC CAC CTG	AGGGCAGAGGCAACGACGCCTGCTG
Exon 6	ACC TAT GTC CAG CTG TGA AGT CTC CAT CTG AC	GGG ACT AAC AGC CGG AGG CAC AG
TNRC6B[b]		
Exon 1	CTGATTGACAAACCTACCCGAAGTCACATGATC	CACGAAATGTCTGAAGCCAGTCTCCATGCTA
Exon 2	GAAATGTCGTCTTGCCCACCACTTACAAGCAT	CAGGAAAAAAAATAAAAGATATCGGATATTCAAGCCAAGACAGT
Exon 3	TGGAAGGATGTCTGAACTGACTCCCAGCT	GTGACTGCAAACAACCCACCTTAATTACTCTACG
Exon 4	GATGTATTTCAAAGACTGTTTCCCAACCCCTCTCT	CCCAGTGGTTTATATAATGAATGGCTTTCCTGGACT
Exon 5.1	GCCCCTTTGAGGGATTAATGGGTAATGAATGC	CCCTTTCCTAGAAGTTCCTTCTTGGACCAGT
Exon 5.2	GGACTAGGAAATTGGAGGAATGTGAGTGGTCA	GTCCCAAGAGTCATTTTTTGACCCAGTTGATTTCTGA
Exon 5.3	GGAGTGTTGGATCTTGGGGTGCAGCTA	GACAATCAGGATGTGTGGGCCTGTACG
Exon 5.4	ACAGGAAGTGAAGTTGGAGGTCAAAGCACTG	CAGAACTTTCCCAATTGCTGTTTTTTGTCTGATCGAC
Exon 5.5	CCTCTTGGAATGAGAATCCCAGCAAGGATC	CCCCATGCTGAAGTGCCATCATCAATGTC
Exon 5.6	CTGCAACACCTAAGGATGAGGAACCCAGT	CATTAAAATGTACTACCAGCATCTGCCATCACTAGCAT
Exon 6.1	CCACCTTCACCCCACTGGATATTATGGCAT	CCTCAAAAATGAAAAGCTTCACACTTACTAGGCTTCATG
Exon 6.2	CTTGGGGTGAGCCAAATGAAAGCAGT	GATCCCTCAAAAATGAAAAGCTTCACACTTAC
Exon 7.1	GAGATGGAGTAGATAAAATTACAAAGACACGTAAATGCTGCA	GAATGAGCAAGTGCGAACATTTCTAAGCAGGCTA
Exon 7.2	GAGTCTGGAACACCACTGGCTCTCAG	CCTTACTTAGGACCTTGAGGACCCCAGT

Exon 8	CTCAAGTCTACATTTTCAGTCTGTATTAATAAAACTCAG	CTATCCTCCTAAGGAAAAGGACAAAATGACTACACAT
Exon 9	GTAGTAAGACGTGGCTCTCAGGTGAATAATGC	CCTATCCCTAATCAAATTGGGCAGGAGATGC
Exon 10	CCATGAGGATAGTGGGGCAGGTCAC	CAAAAGACAAATGGGAACCAAAGCGGTTCTCATG
Exon 11	GTTTATTGCTAGTGTTTCTCTCTTTTCCCTTCTTGCAT	CAAATTTCCCAAGACAGACAAATGATGCAATAAACTGCA
Exon 12	GAACACAGCCTGTGATTCTTCTGAGGGATC	GATTTGAGATTTTGTTCTAAAAGTCTTCATCTTTGGCAGCTAG
Exon 13	AGATTCCTTTTCTTTTCTTTCTTTTTTATTTTTTTGGCTAG	GCAAATGACCTTTTTAGCCACAGGAAGA
Exon 14	GCACAGACCCTTTAGGTGTCCTCAGT	GCCCGGTTCTTTGCCATCAGAGGCTA
Exon 15	CTGATGGCAAAGAACCGGGCACAGTC	GTGCAACCCCTACCACAGGCCTAG
Exon 16	GTCTTTGAATGACACTTGAAGGCATTCCTGGACT	CCAAGTTCACTGCTACCACCCTCAATCATG
Exon 17	CCATCAGCTTCAGAAGACTGTTTGCATTCCAG	CTTAGTAACATGAATCACAAAAGCAATGTATTCTGAACCTGA
Exon 18	TTCCTTGTCTCAGAATTCCAACCAGGAGTGC	CTTTTAGGTACCAGCAAGTACAAATGAAGTCACAAACTGA
Exon 19	CTCTCAAGGTTTCACTGGTTATACTGACATTTATCTTCAG	GCTGTATCTGGCTCATCCTTCCTGAGACT
Exon 20	GTAGTCACTGAAAATTTTATGTTCGTTATGCTCTAAGAACAGT	CTTTTCCTCTACCAGAAGAAAAAGCAGACAACCATG
Exon 21	GCATAAAAGTGCATAGGAGTCAGGGACCAGT	CCCAACCTTTCCCTCTCTCTTAATACAATCAAGTC
Exon 22	CAAAATGGGCACACTAGGTCTCAGCCAGT	CAGCTGCCAGAATACTCACGGGTGCA
Exon 23	TATGCTTTAGGATTTTCCTGAGATCCATAGCTC	TTAGTGCTGCTGCTGTTCCAAAAAAGGTCA
Iso3 Exon 3	CACAGTATCTGTGGCCTTATTGAGAGAATTTCAGTCT	CTCTACTGTTCAATTTCTACCCAAACTGTACCTTTCAG
Iso3 Exon 4	CAG TGA AAC GGC CCT GTG GTT CTG CA	CCA ACG TAT ACA TCT CTG ACA AAC ACG CTT ATC AG

[a]Amplification was carried out in 20 µl reactions containing 10-20 ng of DNA, 1×FastStart buffer (Roche), 1.5 mM $MgCl_2$, primers at 0.5 µM, and dNTPs at 200 µM (final concentrations), with 0.75 U of FastStart Taq (Roche). PCR consisted of an initial incubation at 94/95°C for 6/8 min, followed by 35 cycles of denaturation at 94/95°C for 30 s, annealing for 30 s, and extension at 72°C for 30 s, with a final extension for 10 min. The annealing temperature used was 63°C, except for the following amplimers of *TNRC6B*: 6.2, 59°C; 8, 60°C; 11, 62°C; 13, 58°C; 23, 60°C.
[b]*TNRC6B* exon numbering is based on Uniprot Q9UPQ9 isoform 1 (1833 amino acids, 23 exons). The two additional coding exons that occur in isoform 3 are numbered according to their position in that isoform and shown at the bottom of the list.

Table S2. Markers used to determine haplotypes surrounding the c.4670C>T variant present in three families

**Marker Name**	**Marker type**	**Genomic location**[a]	**Primer pair 5'-3'**
Tcfms1	Microsatellite	42544517 bp	CTTGAACCCAGGAAGTCAAGGCTGCAGT<br>6-FAM CAAGGAATGACTTCCCCACTTTTTACTACACAG
Tcfms2	Microsatellite	42433855 bp	GTCCTGAAACTACGGAATTCATTCATTAGTTCTAC<br>6-FAM GGCAAGAAAAACAGAGGCTTCCAAGTCAGA
Tcfms3	Microsatellite	42433133 bp	TGTCAAGGTTTTCCCAATGTTTTCTTCGAGGA<br>6-FAM CGGGAAAGGATAATCTGTTCAATAAACAGTACTG
Tcfms4	Microsatellite	42390888 bp	TCAACTCTGTCCCACAGGCCTGTCTG<br>6-FAM TCCAGGGAGGGCAGTGAAAATACCTCAC
Tcfms5	Microsatellite	42775494 bp	CTCACAGTGGGCGTGGGCTGCT<br>6-FAM CTGGCAGCCTCACATTCCTCTTATCCA
Tcfms6	Microsatellite	42782403 bp	CACTGCACTCTGGCCTGGGTGATG<br>6-FAM GGTTCTGTGATTGTCATGATAGGGCCTCAG
Tcfms7	Microsatellite	42939056 bp	ATGCCACTTGATCTAGATAGCAAGATGACTAG<br>6-FAM AGACTTGCCAGACCCCACAATCTGTGA
Tcfms8	Microsatellite	43053571 bp	GGGGAGGCTAAGGGAGGTCTATAGATC<br>6-FAM CTACAGGCCAGTAGGAAACCATTCATCTCTG
rs5758652	SNP	42612408 bp	TGTGGAACCCATATGGGCTGAGTGTC<br>ATTTCTCTGCTTTCATGTATTAGGGTGATCACTG
rs16986035	SNP	42602139 bp	GATGTGCATATGTGTATGTGACCATCTGACAGTGT<br>TTGAACTCCTGACCTCAGGCGATCTGC
rs4453786	SNP	42563308 bp	AAATGGGAGGACTTCTGTGTCGACCTCAG<br>CCCCACTTTGCTCTGATCCCTTCACTG
rs2899354	SNP	42554409 bp	CAGGGATCCTCCGTGTCCCACTGT<br>CACAAAAACCTGCAAAGGGATGTTTACAGCAGCT
rs1548304	SNP	42691488 bp	GGCAAGGGACCTGAAGACCCAGTGAT<br>CAGCGGTGGGGAGTGTGGAGTGA
rs6002674	SNP	42694220 bp	GCATCACCACTGTCTCCATTTCTCACATGTC<br>CAACAGTCGGCCCTTTGGGTCAATCG
rs11704558	SNP	42695148 bp	CCTTAGAAGCTGTGGACCAACTGAAACAGAG<br>CCCCAGCCAGACATGGCCTCAC
rs6002676	SNP	42697216 bp	GCAGAGCATGGCATCTGCACTTACCAG<br>GCGTGGCACAGAGTAAGTGCTCAGCA

[a]Locations for microsatellites refer to the first nucleotide of the repeat.

Table S3. Amino acid sequence altering variants of *TNRC6B* found in 341 ASD samples, comparison with controls, and family follow-up

Nucleotide change[a]	Amino acid change	Number of heterozygous ASD samples/total sequenced[b]	Number of heterozygous control samples/total sequenced[b]	Exome Variant Server (EA) expressed as rare/common alleles (selected AA frequencies in brackets)	Family follow up	PolyPhen 2 prediction
c.46G>A [3]	p.V16M	64/293 [5]	75/379 [6]	875/7515	-	Benign (0.000)
c.108A>C	p.K36N	1/334	1/383	0/7620	Absent in affected sibling; transmitted by parent.	Probably damaging (0.981)
c.622A>G [1&2]	p.T208A	1/335	n/a	0/8350 (25/3947)	-	Benign (0.000)
c.871G>A [1&2]	p.D291N	1/332	1/381	7/8251	Present in one affected sibling (PDD), absent in another; present in two unaffected siblings; transmitted by parent.	Possibly damaging (0.682)
c.875G>A [1&2]	p.R292K	2/332	0/381	2/8250	Absent in affected sibling; transmitted by parent; other case (singleton) transmitted by parent	Benign (0.435)
c.962G>A [1&2]	p.R321K	1/332	0/384	0	Present in affected sibling; transmitted by parent	Possibly damaging (0.932)
c.1511C>T [1&2]	p.S504F	1/330	0/384	0	Transmitted by parent	Probably damaging (0.996)
c.2750G>C [1&2]	p.G917A	1/335	n/a	22/8222	-	Probably damaging (0.997)
c.2911T>C [1&3]	p.S971P	1/335	n/a	1/8247 (55/3887)	-	Benign (0.005)
c.3043G>A	p.E1015K	1/335	0/384	0	Present in one affected and one unaffected sibling; transmitted by parent	Possibly damaging (0.878)
c.3359C>A	p.P1120Q	1/334	0/382	2/8196	Not present in the one parent tested	Probably damaging (0.999)
c.4098delG; 4101A>C	p.I1368fs	1/333	0/383	0	Not present in the one parent tested	n/a

c.4318_4320del	p.I1440del	1/332	0/383	0	Transmitted by parent, affected sib untested	n/a

[a]1&3 = isoform 1 and 3 only; 1&2 = isoform 1 and 2 only; 3 = isoform 3 only. Where no superscript indicated, exon is represented in all 3 isoforms and numbering is given for isoform 1.

[b]The number of samples from each panel found to harbour the variant is shown next to the number of samples successfully screened. Numbers in square brackets refer to homozygous changes. n/a, not available.

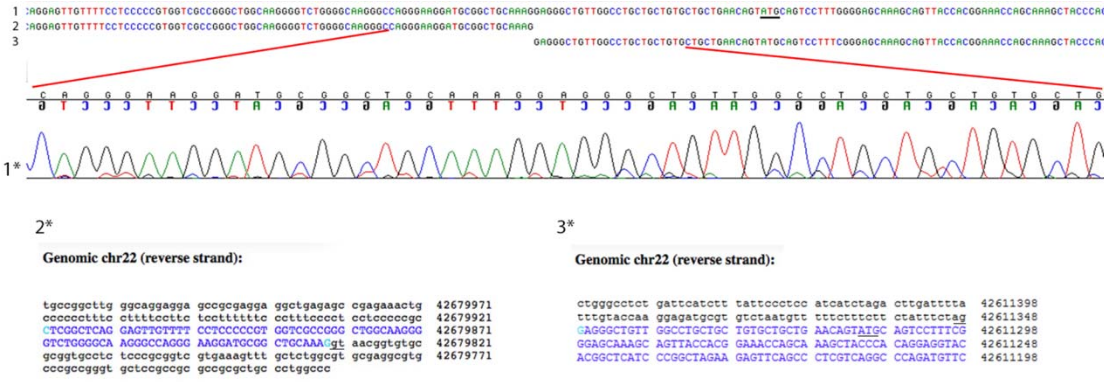

Figure S1. The *TCF20* transcript contains a previously unannotated first exon that encodes an alternative untranslated region. The upper panel shows alignment of sequence obtained via Sanger sequencing of a PCR product amplified from cDNA (1) with sequence from a genomic location ~68,400 bp 5′ of the previously annotated first exon of *TCF20* (2) and the region of the first exon which shows contiguity with this sequence (3). The chromatogram from sequence 1 is denoted 1* while the genomic location of the novel first exon is labelled 2* and the 5′ region of the previously annotated first exon is denoted 3*. The location of the identified exons within the genomic sequence are shown in 2* and 3* denoted by uppercase blue lettering. Note that there is a canonical GT splice donor immediately 3′ of the novel exon 1 sequence (lower case underline in 2*) and an AG splice acceptor 5′ of the exon 2 sequence (lower case underline in 3*). The ATG encoding the initiating methionine is also underlined (upper case lettering) in the upper and lower-right panels.
